# *Alliumnegianum* (Amaryllidaceae): a new species under subg. *Rhizirideum* from Uttarakhand Himalaya, India

**DOI:** 10.3897/phytokeys.183.65433

**Published:** 2021-10-15

**Authors:** Anjula Pandey, K. Madhav Rai, Pavan Kumar Malav, S. Rajkumar

**Affiliations:** 1 Division of Plant Exploration and Germplasm Collection, National Bureau of Plant Genetic Resources, New Delhi 110012, India National Bureau of Plant Genetic Resources New Delhi India; 2 ICAR-National Bureau of Plant Genetic Resources, Regional Station Bhowali, Niglat 263132, Nainital, Uttarakhand, India ICAR-National Bureau of Plant Genetic Resources Nainital India; 3 Division of Genomic Resources, ICAR-National Bureau of Plant Genetic Resources, New Delhi 110012, India ICAR-National Bureau of Plant Genetic Resources New Delhi India

**Keywords:** *Alliumnegianum*, India, *Rhizirideum*, Seasoning spice, Uttarakhand

## Abstract

A new species, *Alliumnegianum* (Amaryllidaceae), belongs to the genus Alliumsubg.Rhizirideum, sect. Eduardia is described here from the Uttarakhand Himalayan region of India. This taxon grows in Malari region of Niti valley in Chamoli district and Dharma valley of Pithoragarh district, Uttarakhand, India. It is a narrowly distributed species and morphologically more closer to *A.przewalskianum* Regel but differentiated by its tunic color of bulb, umbel with lax flowers, peduncle length, perigone colour, size and shape and leaf anatomy. Taxonomic delineation and relationship analysis based on nuclear ribosomal Internal Transcribed Spacers (ITS) region indicated that *A.negianum* is distinct and related to *A.przewalskianum*. This study provided a comprehensive description and comparison with *A.przewalskianum*, an identification key and notes on the distribution of the species.

## Introduction

*Allium* L., one of the largest genera in the family Amaryllidaceae, has about 1,100 species distributed world-wide (Li et al. 2010; Govaerts et al. 2021). The genus *Allium* naturally occurs in dry seasons in the northern hemisphere and South Africa (Friesen et al. 2006; Nguyen et al. 2008; Neshati and Fritsch 2009). The primary centre of evolution for the genus extends across the Irano-Turanian bio-geographical region, and the Mediterranean basin and western North America are considered as the secondary centres of diversity (Friesen et al. 2006). The genus is characterized by bulbs that are enclosed within the membranous or fibrous tunics, free tepals, often a subgynobasic style and well-known characteristic plant odour and taste due to the presence of cysteine sulphoxides (Friesen et al. 2006). The classification of global species in the genus *Allium* is based on molecular phylogenetic analyses, which includes 15 subgenera and 56 sections (Friesen et al. 2006). The Indian *Allium* includes over 10 subgenera, 22 sections and 35–40 taxa excluding cultivated species distributed in different eco-geographical areas of the temperate and alpine regions of Himalayas sharing many taxa of Chinese origin (Pandey et al. 2008, 2017; Li et al. 2010). Indian Himalayan region has two distinct centres of diversity, the western Himalaya (over 85 per cent of total diversity) and the eastern Himalaya (6 per cent), covering the alpine-sub temperate region (2500–4500 m a.s.l.) (Gohil 1992; Pandey et al. 2008).

Globally Alliumsubg.Rhizirideum (G.Don ex Koch) Wendelbo s.str. has ca. 37 taxa that are included in four sections distributed mainly in Europe-East Asia, in China (Friesen et al. 2006; Choi et al. 2012; Jang et al. 2021) and also in Russia, Mongolia and Kazakstan (Sinitsyna et al. 2016; Friesen et al. 2020). *Alliumsenescens* L. of sect. Rhizirideum a species native to northern Europe and Asia from Siberia-Korea and also naturalized in parts of Europe, is an exception (Xu and Kamelin 2000; Li et al. 2010).

Taxa of the subg. Rhizirideum belong to the third and the most advanced evolutionary line, which is phylogenetically sister to taxa of the subg. Allium L., *Cepa* L., *Reticulatobulbosa* (Kamelin) N.Friesen and *Polyprason* Radic. (Friesen et al. 2006; Memariani et al. 2007; Li et al. 2010; Choi et al. 2012). The sect. Eduardia N.Friesen of the subg. Rhizirideum is mainly distributed in the western Himalaya with Pakistan on the west and Nepal and Tibet in the centre, and southwest China on the eastern side. Its habitat mainly comprises of mountainous, snow peak grassland, dry or rocky places in forests, subalpine meadows, steppes, sunny, saline areas, sandy deserts, stony and gravelly slopes, rocky crevices along the stream banks and damp places (Fritsch and Friesen 2002; Choi and Oh 2011; Choi et al. 2012).

Despite the importance of the genus *Allium* for the Indian region, meagre comprehensive studies have been attempted pertaining to molecular and taxonomic evaluation that led to gaps in the status of interspecific and infraspecific relationships among the taxa. Meagre taxonomic studies on the native taxa, unavailability of material for research, sporadic collections from under-explored/unexplored areas and lack of the published literature have led to the possibility of finding new taxonomic records from the Indian region (Pandey et al. 2008, 2017, 2021).

The subg. Rhizirideum is the smallest subgenus of Allium as per the flora of India, and it is represented only by the sect. Eduardia containing only one species, *A.przewalskianum* Regel. This taxon occurs in the scrub, drier slopes, ravines and rocky crevices (2000–4500 m a.s.l.) in Leh, Jammu and Kashmir and Spiti in Himachal Pradesh. The taxa under subg. Rhizirideum are characterized by the presence of several narrowly ovoid-cylindric bulbs, which borne on creeping rhizome usually covered with a common reticulate membrane, leaves shorter than scape, adaxially channeled and stamens slightly longer than perigone segments, spathe with a long beak, nearly 2 to 3 times longer than the base and hemispherical umbel. Most species share a basic chromosome number of *x* = 8 and *2n* = 16 or 32. Occurrence of a polyploid complex in different sections of the subgenus Rhizirideum indicated recent origin of taxa as supported by phylogenetic and biogeographical evidences (Li et al. 2010). Areas with geographical isolation are the driving force of underestimated speciation (Seregin et al. 2015).

A new taxon, *Alliumnegianum*, was collected from the Indo-Tibetan border area of Malari village, Niti valley of Chamoli district in Uttarakhand (India) in 2019 and identity was confirmed by the authors. It is distinct from its closest relative, *A.przewalskianum* Regel (Table 2), the only taxon of subg. Rhizirideum, sect. Eduardia in India. It is characterized by finely reticulated red-brown outer tunics, hemispherical umbel having lax flowers, spathe with a very long beak, deep purple tepals, asynchronous flowering and inner stamen filaments having longer and sharp teeth. In the present work, *A.negianum*, is described and illustrated here. Authors have examined the evidences from morphology, eco-geography, leaf anatomy, molecular study, and taxonomic delineation from other related species.

## Materials and methods

### Taxon sampling and morphological descriptor

A total of 110 plants representing 7 accessions of the new species were collected from the type locality and farmers’ fields in the Niti region of Uttarakhand, India. For delimitation of the taxon with other related species, plants were grown in the Field Gene Bank (FGB) at the ICAR-National Bureau of Plant Genetic Resources (ICAR-NBPGR), Regional Station Bhowali (Nainital), Uttarakhand for comparative study of morphological characters. Data were recorded using the *Allium* descriptor with modifications from the published literature. The floral characters were measured with separate parts to the nearest ten points of the decimal. The seeds having uniform size and maturity were recorded for ultra-features of the characters using the Stereozoom Microscope (LMI, England, model no. SZM167), and the images were captured as JPEG. Ten replicate voucher herbarium specimens of the new species were prepared as per standard procedure and deposited in the National Herbarium of Cultivated Plants (code-NHCP) (Holotype) and CAL (Isotype).

The new species was compared with its closest relative using data derived from the study of specimens preserved in the herbaria of CAL, DD, E, K and NHCP and available literature. Due to its closer affinity with *A.przewalskianum*, all the specimens from diverse sources were critically examined. Taxonomic description and identification key were provided for *Alliumnegianum* and affined species.

### Leaf anatomy

**Figure 1.**
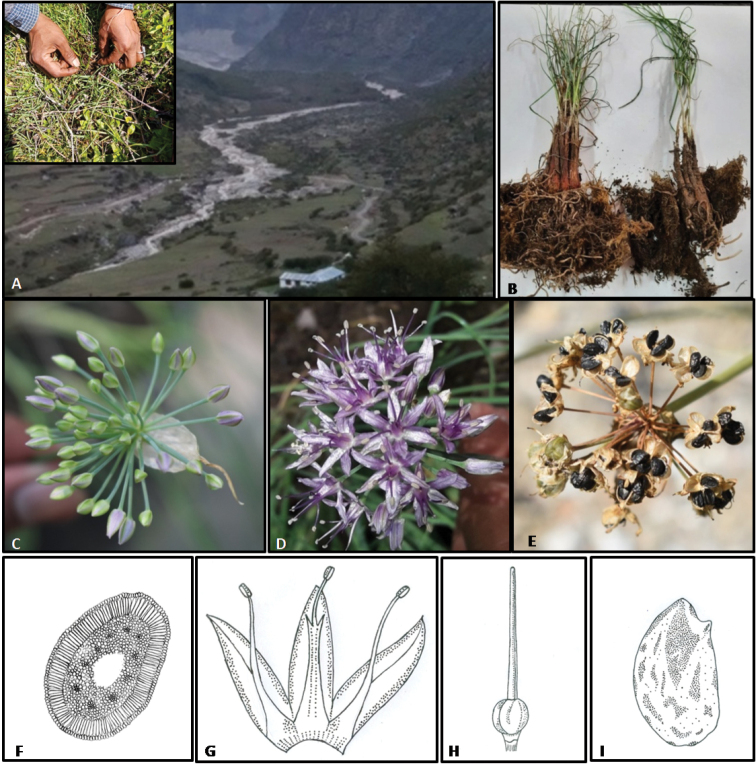
*Alliumnegianum***A** general habitat **B** bulb covered with reticulate fiber on bulbs of *A.przewalskianum* (orange-red) and *A.negianum* (red-brown) **C** inflorescence and spathe with a very long beak, persistent **D** inflorescence **E** capsule with mature seeds **F** line-illustrations of transverse section of leaf showing hollow channel **G** longitudinal section of flower with stamen with two sharp teeth **H** ovary **I** seed with prominent beak (**C–I** magnification × 30–40).

For leaf anatomy live plants were grown in the FGB at Regional Station Bhowali (Nainital), Uttarakhand. Leaf-blades were taken from a point 3–4 cm above the sheaths and fixed in 70% alcohol. Cross-sections were made at three different lengths of leaf and stained with Sartur solution (a mix of sudan III, aniline, chloral hydrate, lactic acid, iodine), the structure was studied, and analyzed with the help of a light microscope (Olympus BH-2) and line diagrammes drawn. The outlines of cells were diagrammatically depicted (Fig. 1F).

### Taxonomic delineation and relationship analysis

#### DNA extraction, amplification and sequencing

Genomic DNA of nine known species and one new taxon (Table 1) which was collected from western Himalayan region and maintained as live material at Field Gene Bank (FGB), ICAR- National Bureau of Plant Genetic Resources, Regional Station, Bhowali, was isolated from fresh leaves using spin column-based Qiamp DNA kit according to the suppliers’ protocol. Selection of taxa for this study was mainly based on the fact that all taxa belong the third evolutionary line representing same eco-geographical areas, and were known by similar local names. This has resulted in confusion of their identity in Indian literature. The quantity and purity of the isolated genomic DNA was tested using the spectrophotometric method. The universal primers ITS1 and ITS4 (White et al. 1990) were used to amplify the ITS regions. The PCR protocol was run at 94 ° C for 5 minutes; 30 cycles of 94 °C for 45 seconds, 55 °C for 45 seconds and 74 °C for 45 seconds and 74 °C for 5 minutes. PCR products were purified using Zymo DNA concentrator kit following the supplier’s protocol. The purified PCR product was used in ABI 3730 DNA sequencer (Applied Biosystems) for generating sequences using PCR primers as sequencing primers. For remaining species from subgenus Rhizirideum and other related sub-genera, the ITS sequence were used from NCBI database.

**Table 1.** List of *Allium* taxa used to generate nuclear ITS sequence in the study.

S. no.	Taxon name	Subgenus	Section	NGB accession number	District; state
1	*Alliumtuberosum* Rottler ex Spreng.	*Butomissa*	*Butomissa*	IC353524	Almora; Uttarakhand
2	*Alliumstracheyi* Baker	*Polyprason*	*Orioprasum*	IC567645	Pithoragarh; Uttarakhand
3	*Alliumprzewalskianum* Regel	*Rhizirideum*	*Eduardia*	IC632207	Leh; Jammu and Kashmir
4	*Alliumnegianum* sp. nov.	*Rhizirideum*	*Eduardia*	IC258493	Chamoli; Uttarakhand
5	*Alliumsativum* L.	*Allium*	*Allium*	IC278243	Chamoli; Uttarakhand
6	Alliumampeloprasumvar.ampeloprasum	*Allium*	*Allium*	IC353526	Pithoragarh; Uttarakhand
7	Alliumcepavar.cepa L.	*Cepa*	*Cepa*	IC410711	Uttarkashi; Uttarakhand
8	Alliumcepa L. var. aggregatum G.Don	*Cepa*	*Cepa*	AP/RP/2014	Chamoli; Uttarakhand
9	*Alliumoschaninii* O.Fedtsch.	*Cepa*	*Cepa*	AP/2014	Voucher; Uttarakhand
10	*Alliumschoenoprasum* L.	*Cepa*	*Schoenoprasum*	IC632213	Kargil; Jammu and Kashmir

**Table 2.** Major morphological characters* (discriminating characters in **bold**) of *Alliumnegianum* in comparison with *A.przewalskianum*.

Character	*A.przewalskianum*	*A.negianum*
Habitat	Carbonaceous slates-gravel; 3300–5200 m	Grassy meadows, open sandy slopes, along rivers/ streams; 3000–4800 m
Plant habit	Erect	Semi-erect
Plant growth (under experimental condition)	Robust, shorter	Taller, plants and leaves
Plant height (cm)	20–45	27–50
Bulbs no. in cluster	2–4	2–7
Bulb no., shape	Cluster 3–4; cylindrical-narrowly ovoid	Cluster 4–8; cylindrical-narrowly ovoid
Bulb length (cm)	10.2–12.5	6.8–12
Bulb diameter (cm)	0.6–0.7	0.8–1.2
Tunic outer**	Finely reticulate; **reddish-orange-brown**	Finely reticulate; **reddish-dark brown**
Tunic inner	Membranous, brown-red	Membranous, orange-red
Rhizome type; size (mm)	**Vertical, short; 3**–**5**	**Oblique; 7**–**12**
Leaf no., colour	3–5, lighter brown-green	4–6, dark green
Leaf *vs.* scape	Much shorter than scape	Slightly shorter than scape
Leaf blade shape; apex	Linear, not fistular; obtuse to subrounded	Linear, filiform; acute
Leaf length (cm)	15–30	12–40
Leaf width (mm)	2.0–2.5	1–3.2
Leaf erectness	**Erect**	**Erect**-**semierect**
Leaf waxiness	Non-waxy	Waxy
Leaf cross section	Circular	Circular
Spathe valve if persistent	1(2)-valved, persistent	1-valved, persistent
Spathe valve shape, size	Ovate	Ovate-oblong
Spathe size (cm)	2–3 (two times the base; short, blunt)	4–6 (long narrow beak; 3 times the base)
Scape type	Solid, terete, erect, central	Solid, terete, erect to semi-erect, lateral-central
Scape size (cm)	30–40 × 0.2–0.35	20–50 × 0.36–0.48; 1/3-of the base
Pedicel *vs.* perigone	Subequal	2–3 times longer
Umbel flower opening pattern	**Synchronous (80 per cent)**	**Asynchronous (30**–**40 per cent)**
Umbel shape	**Spherical-hemispherical, densely flowered, compact**	**Hemispherical, lax, loosely flowered**
Umbel diameter (mm)	28.5–30.2	25.1–42.0
Umbel flower (no.)	25–40	30–40
Peduncle size (cm)	0.5–1.0	0.8–2.5
Flower size (cm)	0.4–0.5×0.3	0.5–0.5× 0.4
Flower color	**Pale red-purple pink (variable)**	**Dark purple (as recorded now)**
Perigonium shape and color	Campanulate, **pink-dark purple, tepal wide open**	Campanulate, **lilac, light to dark purple, tepal partly opened**
Tepal shape	**Ovate**-**lanceolate**, apex obtuse	**Elliptic**, **ovate-lanceolate**; apex-acuminate-mucronate
Tepal inner size length × width (cm)	0.3–0.4 ×0.2–0.3	0.5–0.6 × 0.3–0.4
Tepal outer size length × width (cm)	0.5–0.7 × 0.2–0.3	0.6–0.7 × 0.3–0.5
Tepal apex shape	Acute-acuminate	Acute, mucronate
Tepal maturity	Curved outwards	Slightly inwardly curved/rolled
Tepal mid-vein	Non-conspicuous; purple green-dark purple	Very conspicuous; green-light green
Anther length (mm)	6.1–9.3	6.8–8.5
Anther lobe length (mm)	Oblong-ovate, 1–2	Oblong, 1–2
Anther lobe color	Yellow-purple	Yellow-purple
Filament color	Yellowish-purple	Greenish yellow-purplish green
Filament length, position	Double the size of tepal; exserted,	Half the size of tepal; slightly exerted
Filament inner and outer anther	Inner – **two sharp teeth up to 1/2 to 1/4** length of filament with broader base; outer narrower base	Inner – **two shallower-sharper teeth up to 1/2 to 2/3** length of filament with base as wide as tepal; outer narrower base
Ovary shape	Ovoid – globose, wrinkled	Obovoid – subglobose
Ovary color	Purple green, tinged with purple	Dark-pale purple
Ovary style *vs.* anther (after pollination)	**Much exserted**, longer than the ovary	**Slightly exserted** or equal
Ovary stigma tip	Acuminate-acute	Acuminate
Stigma *vs.* stamen	Sub-equal	Slightly longer
Capsule shape	Ovoid	Sub-globose
Seed length (mm)	2.75–2.96	3.21–4.05
Seed width (mm)	1.55–1.59	1.92–1.97
Seed color	Dull black	Shiny black
Seed no./locule	2	2
1000 seed wt (g)	2.12	2.73
Odour when crushed#	Strong onion-light garlic	Strong onion-garlic

*data recorded from a minimum of 30 specimens of each taxon; additional 43 e-images; **: recorded immediately after uprooting; #: data on feedback; also refer Pandey et al. 2021.

#### Phylogenetic analysis based on the comparison of sequences

The generated DNA sequences from both the primers were checked for alignment using the BioEdit software. Multiple pairwise alignments of generated sequences and from NCBI database were made using ClustalW. The aligned sequences were used to generate the genetic distance between taxa and the evolutionary history, which was inferred by using the Maximum Likelihood method based on the Jukes-Cantor model using MEGA7.0 (Kumar et al. 2016).

## Result and discussion

### Taxonomic treatment

#### Allium negianum

Taxon classificationPlantaeAsparagalesAmaryllidaceae

A.Pandey, K.M.Rai, Malav & S.Rajkumar sp. nov.

040A06F2-2C03-5ECB-9E99-47717BA649B0

urn:lsid:ipni.org:names:77220799-1

Figs 1 , 2

##### Type.

India, Uttarakhand: Chamoli, rocky areas (altitude 3000–4800 m), 22 Aug. 2019, KMR/AS/02/19 (Holotype: NHCP; Isotype: CAL; Seeds conserved in the National Genebank, New Delhi: IC258493).

##### Description.

Herbs, hermaphrodite, 27–50 cm tall. Rhizome condensed, 6.5–8.5 mm long, oblique. Bulb clustered, cylindric to narrowly ovoid, 0.8–1.2 cm in diameter, 6.8–12 cm long, outer tunic finely reticulate, reddish-dark brown, inner membranous, light-brick red. Leaves 4–6, slightly shorter than scape, 12–40 cm × 1.0–3.2 mm, erect, to semi-terete to terete, dark green; base slightly bulbous. Scape terete, semi-erect, covered with leaf sheaths at base only, stout, solid in cross-section (hollow in mature), 15–30 cm × 3.5–5.5 mm. Spathe 1-valved, persistent, beak very narrow-long, 2.5–4 mm. Inflorescence umbellate, hemispheric, 30–40 lax flowered. Peduncle subequal, 16–18 × 2–3 mm, without bulbils. Flowers bisexual, perigone campanulate, tepals dark purple with distinct green mid-line; inner tepals slightly longer than outer ones, oblong-lanceolate, apex acute, 6–8 × 3–4 mm; outer segments ovate to narrowly so, 5.5–6 × 2.5–3 mm. Stamens anthers oblong, yellow-purplish (on maturity), 2.3–2.6 mm long; filaments subequal, 6.8–8.5 mm, purple, slightly exserted, connate at base and adnate to perigone segments; outer ones subulate; inner ones broadened for 1/2–1/4 to their length, one sharp toothed on each side. Ovary sub-globose, purple-tinged, 3.6–4.8 × 1.8–3.5 mm. Style terete, exserted, stigma smooth, acute-acuminate, ovules 2 per locule. Capsules trigonous, 5–5.5 × 5.8–7.2 mm; seeds obovate with a prominent notch on one side, 3.2–4.0 × 1.9–1.9 mm, testa deep black. Plant has strong onion-garlic type aroma.

**Figure 2.**
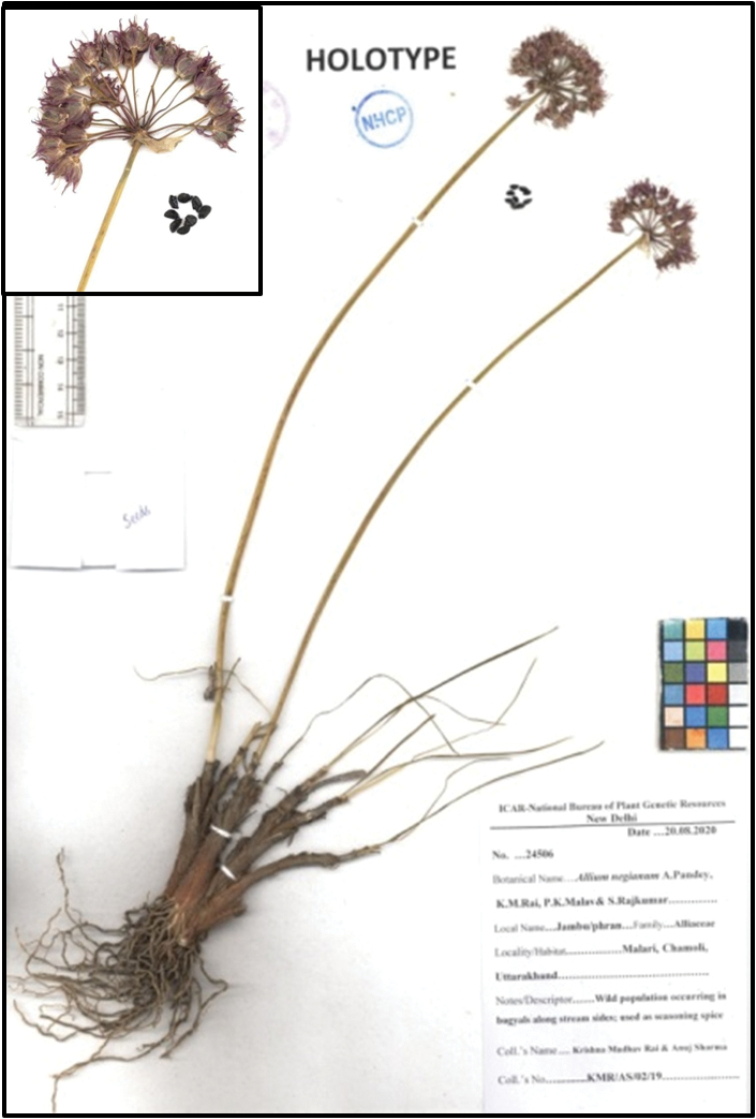
Holotype specimen of *Alliumnegianum* deposited in NHCP.

##### Habitat.

Slopes, sandy soils along rivers and streams along the alpine meadows (altitude 3000–4800 m asl) in Sumna valley (villages Gamsali, Niti, Tolma, Kailashpur and Farkya) in Chamoli district near Malari glacier of India.

##### Etymology.

The specific epithet, “*negianum*”, is named in honour of Late Dr. Kuldeep Singh Negi, an eminent explorer who has dedicated his life in collection of indigenous *Allium* species germplasm along with associated indigenous knowledge across the country. He was also instrumental in establishing the *Allium* Field Gene Bank (FGB) at the Regional Station, Bhowali, Uttarakhand. The entire germplasm of indigenous *Allium* species collected by him from remote areas of the country are characterized and successfully conserved at *Allium*FGB, Bhowali, Uttarakhand.

##### Vernacular/local name.

Pharan, phran, jambu, sakua, sungdung, kacho, etc. (Pandey et al. 2021).

##### Phenology.

Flowering and fruiting is from June to middle September (altitude 3000–4800 m a.s.l.).

##### Leaf anatomy.

The transverse section of the leaf of *A.negianum* showed an elliptical outline. The epidermis has small cells covered with a thin cuticle layer, and stomata are narrowly distributed along the surface area. Single layered compactly arranged palisade tissue comprised of long cylindrical cells. The mesophyll cells are spongy tissue and compact in young leaf as well in the proximal ends of mature leaf while in the centre part of mature leaf, broken mesophyll cells are confused with fistulous leaf appearance; 10–12 vascular bundles are arranged along with the palisade tissue across the entire circumference (Fig. 1F).

##### Seed morphology.

Seed characters and testa sculptures represents a good taxonomic character in *Allium* (Neshati and Fritsch 2009; Celep et al. 2012; Lin and Tan 2017). Apparently, the seeds of the newly described species were marginally bigger than the related taxon, *A.przewalskianum*. Baasanmunkh et al. (2020) have discussed on the seed testa structure and its taxonomic implication for taxa of the subg. Rhizirideum. The seed size in *A.negianum* (Fig. 1I) measured 3.2–4.0 × 1.9–1.9 mm in contrast to 2.7–2.9 × 1.5–1.5 mm in the later taxon (Fig. 1I). The seeds of *A.negianum* are obovate in shape with a prominent notch on one side, gradually concave from edge to centre, with deep black and wrinkled testa.

The testa cell shape was irregularly hexagonal-pentagonal, loose with clear meshes of reticulated tissue. The anticlinal walls are usually raised, prominently small to intermediate granulose verrucae. The periclinal cells wall has several verrucae with irregular depressions. Study indicated that in subg. Rhizirideum testa cell shape varied from oval to irregular or oval to hemispherical; and seed length 1.30–2.35 mm, anticlinal wall were distinguished by nearly S type to straight and periclinal wall was flat to nearly convex with densely granulated verrucae (Baasanmunkh et al. 2020). *A.przewalskianum* was distinguished by irregular testa cells in a loose arrangement with reticulated tissue, straight to arched anticlinal walls, and concave periclinal walls with small to intermediate verrucae and granules (Lin and Tan 2017).

##### Distribution and ecology.

**Figure 3.**
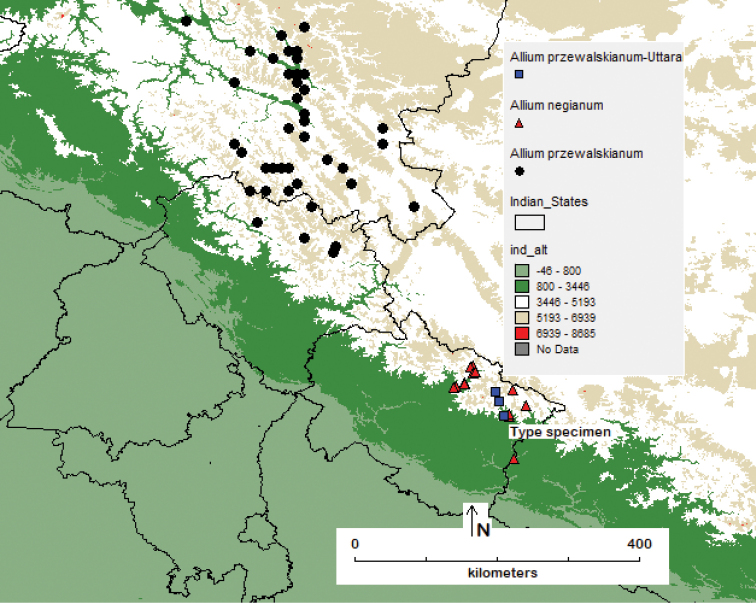
Distribution of taxa of Alliumsubg.Rhizirideumsect.Eduardia in India: *Alliumnegianum* and *A.przewalskianum* shown by the red triangle and black circled dots respectively; blue rectangle showed the occurrence of *A.przewalskianum* as per the data from GBIF (records of occurrence from Uttarakhand).

The sect. Eduardia of the subg. Rhizirideum is distributed in the southern most range of the Himalayan region of India extending to China which is the centre of diversification. *Alliumnegianum* is a species recorded from the southernmost transitional zone between India and China. The distribution of *A.negianum* is restricted to the phytogeographical region of western Himalaya from Sumna valley, Malari, Chamoli district of Uttarakhand, in western Himalaya, India where it commonly occurs along the open grassy meadows, sandy soils along rivers and streams occurring in the snow pasture lands along the alpine meadows (locally known as ‘*bugyal*’ or ‘*bugial*’) between 3000–4800 m a.s.l. (Fig. 1A; Fig. 3) in synanthropic habitats. It was reported growing as wild population in Darma valley of Pithoragarh, along Gori Ganga (also Gori Gad) river in the Munsiyari, Pithoragarh district, in Milam Glacier, in northeast of Nanda Devi, Uttarakhand, India. The seeds flowing with the melting snow led to its broader spread in the areas with good regeneration reported by the authors (Fig. 1A). Hence the taxon may be considered endemic in the area of study. Indiscriminate harvest of leaves and bulbs used for ‘seasoning’ purposes has threatened its wild population.

The first report on large scale cultivation of this taxon in Niti valley, Uttarakhand, as ‘seasoning allium spice’ called ‘*jambu*’ and ‘*phran*’ has been published (Pandey et al. 2021). Though the taxon was reported commonly under cultivation, the authors have observed the wild populations primarily from the above ‘type’ locality. The authors could not trace large scale cultivation of another taxon, *A.stracheyi* (used for same purpose and known by same local name) in the described locality in Uttarakhand (Pandey et al. 2021). Considering that *A.stracheyi* was a rare species reported from wild habitats in Uttarakhand Himalaya, the authors assume that the reports by Kuniyal and Negi (2018) on large scale cultivation may be referring to this newly described taxon which is also known by the same local name. Unfortunately, earlier studies on *A.stracheyi* did not provide any locality details, nor were the voucher specimens deposited in any herbaria of the material used in their study. Therefore, validation of the taxonomic identity could not be ascertained. Also, there is no occurrence record of the taxa belonging to subg. Rhizirideum from Uttarakhand, India.

##### Specimens examined

**(*Paratypes)*.***Alliumprzewalskianum*: India. Himachal Pradesh. Spiti, Takcha 25 Jul. 1972 *U.C. Bhattacharya 48815* (BSD); Tobo, Kinnaur, Lahul & Spity, 15 Sept. 2007, *V.D. Verma & Ramchander* (NHCP); Jammu & Kashmir. Ladakh, 25 July 1941, *Ludlow & Sheriff8529* (BM); 8 Sep.1941, *Ludlow & Sheriff8571* (BM); Ganglas, 1 Aug. 1988, *H.J.Chowdhury & B.P.Uniyal 86043* (BSD); 1880, *Aitchinson376* (CAL); Kashmir. Nubra, 24 July 1980, *A.R. Naqshi & G.N. Dhar7370* under *A.stracheyi*; Leh (J&K), 8 Sept. 2014, *K. Pradheep & P.S. Mehta1733* (NHCP); Leh (J&K), Nov. 2014, *K.Pradheep HS21817* (NHCP); Pangu lake, Luthum village, Leh (4500 m), *s.s. Malik & D. Gautam15298* (NHCP); Uttarakhand. Malari, Chamoli, 10 Sept. 2019, *Badal Singh & K.Madhav RaiHS24013* (NHCP); *Alliumauriculatum*: Uttarakhand: Brahmmathya, district Chamoli, August 1988, *K.S.Negi & M.N.Kopper 9387* (NHCP).

##### Online herbaria.

*A.stoliczki*: Ladakh, Khaedubgla, 18 Aug. 1982, *P.K.Hazra*98623(K), 1985, *Jacquemont V. Type* (K); *T. Thomson*, *Type* (K); China, 1 Jan. 1872, *Przewalski N.M.*, #*s.n.*, *Type (P)*; 01 Jan. 1884, *Przewalski N.M.*, *Type* (P, K); 1872–1873, *Przewalski N.M.*, #*s.n.*, *Type* (G).

There are no records on the availability of this new taxon from Uttarakhand (Dasgupta 2006). Shah (2014) has raised doubts on reported cultivation of *A.przewalskianum* in Uttarakhand by Negi (2006). Also recorded data on the occurrence of allied taxon under *A.przewalskianum* from Gori, Kumaon, Uttarakhand (dated 16 June 2005) and Gori, Martoli, Uttarakhand (7 Oct. 2004) during the study of a total of 413 specimens in the GBIF database need critical study.

##### Note.

*Alliumnegianum* was previously mistaken for identity as *A.stracheyi* as noted in the published records from India. Despite no morphological similarity with the latter taxon, Kuniyal and Negi (2018) referred ‘*phran*’ as *A.stracheyi*. In literature, it was also referred as *A.auriculatum* and *A.przewalskianum* due to morphologically similarity of the outer tunics (Pandey et al. 2021). However, the present study demonstrated that *A.negianum* is clearly distinguished from *A.przewalskianum* and *A.stracheyi*, particularly characters of the bulb tunic color when fresh, umbel, teeth in filament and perigone size and color (Fig. 1B–H; Table 1). *Alliumnegianum* is diploid (*2n = 2x* = 16) (data not produced), whereas *A.przewalskianum* is reported to be tetraploid (*2n = 4x* = 32) as well as diploid with no stated morphological variation except the stout habit. Authors noted that *A.negianum* has robust plant habit, stronger plant aroma in wild habitat as compared to plants growing under cultivation. In contrast, the related taxon of the subg. Rhizirideum is currently distributed in Jammu and Kashmir, Himachal Pradesh and adjoining parts in Nepal. *A.negianum* is reported from areas of Uttarakhand and only known from the type locality (altitude 3200–4800 m a.s.l.) and has never been collected from elsewhere in India and other parts of the world. Therefore *A.negianum* is said to be localized in distribution.

Upon critical examination of specimen of *A.auriculatum* deposited in the NHCP, all plant characters were found to be closer to *A.negianum*. Four specimens of this taxon were noted in label data as frequently growing on flat rocks in Brahmmathya, district Chamoli (3800 m asl.), Uttarakhand, used as leaves cooked as a vegetable.

*Alliumnegianum* is morphologically allied to a Chinese species *A.eduardi* Stearn that occurs on the dry slopes and plains in the adjoining regions of Mongolia and Russia and shares characters of spathe beak size, hemispherical umbel and perigone shape, but differs in having yellowish-brown bulb tunic color, tepal apex with a reflexed point and shorter stamen teeth length.

### Taxnomic treatment

Two species, *A.przewalskianum* and *A.negianum*, of the subg. Rhizirideum, sect. Eduardia can be distinguished from *A.stracheyi* of the subg. Polyprason by using the following key.

### Key to *Alliumnegianum* and related species

**Table:** 

1	Bulbs cylindrical-narrowly oblong-ovoid, outer tunic fibrous, with finely reticulate texture, reddish-dark brown, leaves semiterete-terete	**2**
–	Bulbs cylindric-narrowly ovoid, outer tunic fibrous scarious, brown-darkest brown, leaves narrow, fistulous	***A.stracheyi***
2	Bulbs outer tunic reticulate, reddish, inner tunic membranous, red-orange, rarely light brown; umbel compact globose, tepal pale-red to dark purple; filaments longer than perigone segments, inner ones broadened for 1/3–1/2 their length with shallow teeth; style very much exserted after anthesis	***A.przewalskianum***
–	Bulbs outer tunic reticulate, reddish-brown, inner tunic membranous red; umbel hemi-spherical, lax; tepals dark purple-pink purple; filaments equal to perigone segments, inner ones broadened at the base for 2/3–1/3 of length, sharply marked teeth; style slightly exserted after anthesis	***A.negianum***

### Taxonomic delineation and relationship analysis using nuclear ITS sequence

**Figure 4.**
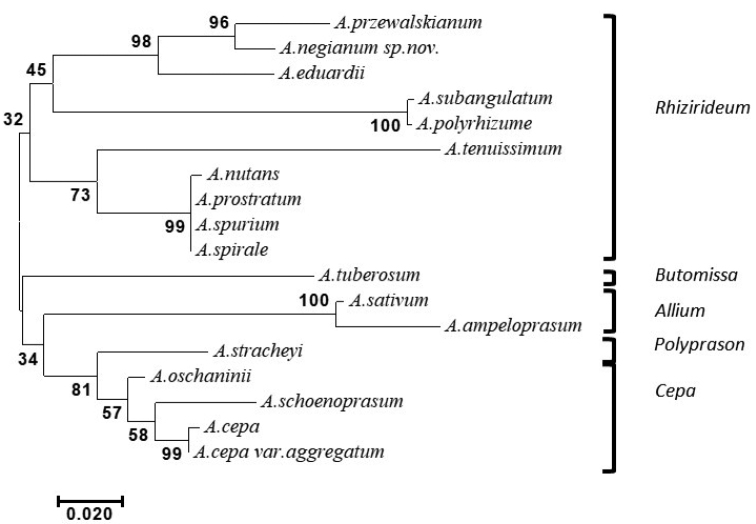
Maximum likelihood tree from nuclear ribosomal ITS sequence from *Allium* taxa showing distinctness of *Alliumnegianum* sp. nov.

For taxonomic delineation and relationship analysis data set comprising 18 representative taxa from diverse subgenera were selected (Table 3; Fig. 4). The DNA sequence data set of nuclear Internal Transcribed Spacers (ITS) region used for phylogenetic analysis was generated for *Alliumnegianum* and other taxa used in the study. The generated ITS sequences and obtained ITS sequences from NCBI (Table 3) were used to construct the maximum likelihood tree. The tree with the highest log-likelihood is shown (Fig. 4). The percentage of trees in which the associated taxa clustered together is shown next to the branches. Initial tree(s) for the heuristic search were obtained automatically by applying Neighbor-Join and BioNJ algorithms to a matrix of pairwise distances estimated using the Maximum Composite Likelihood (MCL) approach and selecting the topology with superior log likelihood value. The branch lengths measured in the number of substitutions per site.

**Table 3.** Details of nuclear ITS sequence used in present study.

Sl. No.	Species	Genbank accession number
1	*Alliumtuberosum* Rottler ex Spreng.	MZ567234 (present study)
2	*Alliumstracheyi* Baker	MZ567226 (present study)
3	*Alliumprzewalskianum* Regel	MZ567224 (present study)
4	*Alliumnegianum* sp. nov.	MZ567225 (present study)
5	*Alliumsativum* L.	MZ567230 (present study)
6	Alliumampeloprasum L. var. ampeloprasum	MZ567231 (present study)
7	Alliumcepa L. var. cepa	MZ567228 (present study)
8	Alliumcepa L. var. aggregatum G.Don	MZ567232 (present study)
9	*Alliumoschaninii* O.Fedtsch.	MZ567229 (present study)
10	*Alliumschoenoprasum* L.	MZ567227(present study)
11	*Alliumeduardii* Stearn ex Airy Shaw	MK917745
12	*Alliumsubangulatum* Regel.	AJ411870
13	*Alliumtenuissimum* L.	AJ411846
14	*Alliumnutans* L.	JN864787
15	*Alliumprostratusm* Trevi.	LN867014
16	*Alliumspurium* G.Don.	LN867017
17	*Alliumspirale* Willd.	JN864784
18	*Alliumpolyrhizum* Turcz. ex Regel	MK917742

Source: S. no. 1–10 refer table 1; 11–18: NCBI

Two major clades were found within *Allium*, comprising subgen. Rhizirideum, on one side and second cluster had four subg. Butomissa, *Allium*, *Polyprason* and *Cepa*. on the other side. This former group was divided in two sister clades, with first clade having *Alliumprzewalskianum*, *Alliumnegianum* sp. nov. *A.eduardii* (all from section Eduardia); *Alliumsubangulatum*, *A.polyrhizum* from sect. Caespitosoprason; and *A.nutans*, *A.prostratusm*, *A.spurium* and *A.spirale* in sect. Rhizirideum. One of the taxon *A.tenuissimum* from sect. Tenuissima grouped separately. Second clade was divided into subgenera, namely *Butomissa* with one taxon, *Alliumtuberosum*; subg. Allium, with *Alliumsativum* and Alliumampeloprasumvar.ampeloprasum; subg. Polyprason having *Alliumstracheyi*; subg. Cepa that was the largest having four taxa, Alliumcepavar.cepa, A.cepavar.aggregatum, *A.oschaninii* and *A.schoenoprasum* from distinct sections.

Based on the likelihood tree, the new *Allium* taxon was observed to be closely related to *A.przewalskianum*, both of Indian Himalayan origin along with a Chinese taxon, *A.eduardii* to form distinct cluster supporting the morphological resemblance of this taxa with section Eduardii under subg. Rhizirideum. The species from other sections under same genus were distantly placed in the phylogenetic tree. The species which are found in same geographical area belong to different subgenera viz. *Allium*, *Cepa*, *Butomissa* and *Polyprason* were distantly placed and used as outgroup in determining the integrity of newly described species *Alliumnegianum*.

The above findings indicated that the new taxon is a distinct species and is closely related to *A.przewalskianum* and belongs to sect. Eduardia under subg. Rhizideum. These findings supported the observations recorded using plant morphology, particularly the floral characters that were very distinct in both the taxa.

Recent advances in molecular phylogenetics have revolutionized our understanding of *Allium* taxonomy and evolution. However, the phylogenetic relationships in some *Allium* sections (such as the Alliumsect.Eduardia) and the genetic bases of adaptative evolution remain poorly understood for the Indian taxa (Pandey et al. 2021). Molecular phylogeny study of the wild *Allium* in different centers of diversity (Nguyen et al. 2008; Xie et al. 2019; Jang et al. 2021) has helped in unlocking many aspects of the taxon relationships. The present study uncovered a new species relationship with its closest allied species and suggested that the selective habitat pressure has played an important role in the adaptation and evolution of *Allium* in this habitat which will facilitate uncover more taxa in the genus.

## Conclusions

*Alliumnegianum*, a new species under the subg. Rhizirideum, is described using live and herbarium specimens. With the inclusion of this taxon, in the subg. Rhizirideum of the sect. Eduardia there are two taxa in India, and the latter one *A.negianum* was reportedly restricted to the Uttarakhand flora. Samples of this taxon collected during earlier explorations that remained unidentified will be designated with this new name and conserved as seed in the National Gene Bank (NGB), New Delhi and vegetative material will be maintained in the Field Gene Bank (FGB) at Bhowali, Uttarakhand, India.

## Supplementary Material

XML Treatment for Allium negianum
